# Response of microbial communities in the tobacco phyllosphere under the stress of validamycin

**DOI:** 10.3389/fmicb.2023.1328179

**Published:** 2024-01-18

**Authors:** Moyan Guo, Jingrong Hu, Chaoying Jiang, Yi Zhang, Hancheng Wang, Xinghong Zhang, Tom Hsiang, Caihua Shi, Qing Wang, Feng Wang

**Affiliations:** ^1^College of Agriculture, Yangtze University, Jingzhou, Hubei, China; ^2^Guizhou Academy of Tobacco Science, Guiyang, Guizhou, China; ^3^Institute of Advanced Agricultural Science, Hubei University of Arts and Science, Xiangyang, Hubei, China; ^4^Guizhou Tobacco Company of China National Tobacco Company, Guiyang, Guizhou, China; ^5^College of Agriculture, Guizhou University, Guiyang, Guizhou, China; ^6^School of Environmental Sciences, University of Guelph, Guelph, ON, Canada

**Keywords:** tobacco brown spot, validamycin, high-throughput sequencing, microbial composition, phyllosphere microorganisms

## Abstract

Validamycin, is classified as an environmentally friendly fungicide. It has high efficacy with little associated pollution risk, and it has been used in China on tobacco for many years especially during leaf spot season. To understand changes in microbial communities and functional aspects of the tobacco phyllosphere after exposure to validamycin, the chemical was sprayed on tobacco leaves during brown spot epidemic periods caused by *Alternaria alternata*, and asymptomatic and symptomatic leaves of tobacco were sampled at different times (0 day before, 5, 10, and 15 days after application). The fungal and bacterial population diversity and structure were revealed using Illumina NovaSeq PE250 high-throughput sequencing technology, and Biolog-ECO technology which analyzes the metabolic differences between samples by using different carbon sources as the sole energy source. The results showed that the microbial community structure of both asymptomatic and symptomatic tobacco leaves changed after the application of valproate, with the microbial community structure of the asymptomatic tobacco leaves being more strongly affected than that of the symptomatic leaves, and the diversity of bacteria being greater than that of fungi. Phyllosphere fungal diversity in asymptomatic leaves increased significantly after application, and bacterial abundance and diversity in both asymptomatic and symptomatic leaves first increased and then decreased. Validamycin treatment effectively reduced the relative abundance of *Alternaria*, *Cladosporium*, *Kosakonia*, and *Sphingomonas* in leaves showing symptoms of tobacco brown spot, while the relative abundance of *Thanatephorus*, *Pseudomonas,* and *Massilia* increased significantly after application. Furthermore, the ability to metabolize a variety of carbon sources was significantly reduced in both types of leaves after validamycin application, and both types had a weaker ability to metabolize *α*-Ketobutyric Acid after application. This study reveals phyllosphere micro-ecological changes in symptomatic and asymptomatic tobacco leaves during different periods after validamycin application and the effects on the metabolic capacity of phyllosphere microorganisms. It can provide some basis for exploring the effect of validamycin on the control of tobacco brown spot.

## Introduction

Tobacco is an economically important crop in China, which accounts for 39.6% of global tobacco production and approximately 40% of global tobacco consumption (Wang et al., 2013, 2014). Many fungal and bacterial diseases occur on tobacco leaves during production (Xu et al., 2014), even during the curing of leaves (Chen et al., 2020). Common diseases on tobacco leaves include target spots, brown spots, wildfire and powdery mildew (Xiao et al., 2003; Wu et al., 2012; Feng et al., 2022). Once leaf spots are prevalent, they can lead to lower-quality tobacco leaves, significantly reduce yields, and cause huge economic losses. For tobacco leaf spot disease management, validamycin is the only agent in China registered for controlling tobacco target spot, caused by *Rhizoctonia solani*. This fungicide has also been reported to have high activity against many other diseases, such as *Rhizoctonia* diseases, rice sheath blight, corn southern leaf blight, and rice false smut (Lueas, 1975; Kazuho, 1985; Asano et al., 1987; Müller et al., 1995). Validamycin is an antimicrobial pesticide isolated from an actinomycete, which interferes with and inhibits the growth and development of fungi after being absorbed by the fungal cells (Masaru et al., 1986; Copping and Menn, 2000). Years of large-scale field application have fully demonstrated its high efficacy, and it is considered harmless, pollution-free, and environmentally friendly for sheath blight disease. However, the effects of validamycin on tobacco brown spot caused by the ascomycete *Alternaria alternata* is not certain, and its effects on phyllosphere microorganisms has not been well studied.

The types and numbers of phyllosphere microbiota are closely related to plant growth development and metabolic activities (Lindow and Brandl, 2003; Leveau, 2015). Host plants and phyllosphere microorganism are in a dynamic equilibrium process (Blacquière et al., 2012; Carrión et al., 2019). Additionally, the microbial structures showed complex dynamic changes as affected by weather, soil, and other environmental factors. The study of Lindow and Leveau (2002) showed that bacterial community diversity in olive leaves was lowest during the warmest and driest months of the year and highest during cooler and wetter months. Carbon sources are the basis of microbial life activities. Biolog ECO metabolic phenotype technology can reflect the metabolic capacity of all microorganisms in the ecological environment to 31 common carbon sources, then reflect the nutritional requirements characteristics of environmental microorganisms, and understand the changes of microorganisms (Liu et al., 2021). Analysis of substrate utilization profiling in the tobacco brown spot revealed that the microbial community was highly utilized by carbohydrates, followed by carboxylic acids, amino acids, polymers and amines, however the utilization of phenolic compounds was minimum (Dai et al., 2022). Exploring the carbon metabolic ability of tobacco phyllosphere microorganisms after the application of the agent can confirm the influence of the agent on the microorganisms.

The population structure of phyllosphere microorganisms is closely related to the occurrence of plant disease (Mejia et al., 2008; Huang Y. et al., 2021). Xiang et al. (2022) found that microflora structure and diversity are different in symptomatic and asymptomatic tobacco leaves. At the early stage of tobacco brown spot, symptomatic leaves of different age had similar sets of dominant fungi and bacteria than the asymptomatic leaves. The dominant fungi included species of *Alternaria*, *Didymella*, *Cladosporium*, and the dominant bacteria included species of *Pseudomonas* and *Sphingomonas* (Xiang et al., 2022). In the middle and late stages of tobacco brown spot development, *Pantoea* was abundant in the bacterial community (Dai et al., 2022). The dominant phyllosphere fungi associated with leaves bearing tobacco brown spot were *Alternaria*, *Phoma*, *Fusarium,* and *Cladosporium* (Liu et al., 2009). In addition to the pathogenic fungus, *Alternaria*, the cause of tobacco brown spot, there are also species of *Phoma* and *Fusarium* associated with leaves. Whether these pathogen also cause tobacco leaf disease or increase the incidence of tobacco brown spot still need to be further investigated.

The composition of fungi and bacteria of tobacco brown spot diseased leaves have been widely reported (Dai et al., 2022). The occurrence of diseases changes the structure and diversity of the microbial community, and the composition of the microbial flora also is change by the host leaf maturity, the environment, and the use of control agents (Miller et al., 2018). The study of the changes of phyllosphere microorganisms can confirm the effect of agents on diseases. In tobacco leaves, the application of low fungicide dosages can significantly affect the population structure of beneficial bacteria such as species of *Stenotrophomonas*, *Serratia*, and *Flavobacterium*, which are known to be beneficial for distinct agricultural plants (Chen et al., 2020). When conditions are conducive to tobacco target spot caused by *R. solani*, the application of azoxystrobin can reduce disease incidence, and also cause significant changes in the diversity, community composition and relative abundance of both target and non-target microbes on asymptomatic and symptomatic tobacco leaves (Sun M. et al., 2023). Dimethachlon, a dicarboximide fungicide, can significantly affect the change of the population structure of beneficial bacteria such as species of *Stenotrophomonas*, *Sphingomonas*, *Flavobacterium*, and *Serratia* in tobacco leaves (Chen et al., 2021). Validamycin can provide effective control of *Alternaria*, which was tobacco brown spot diseased pathogen (Aftab et al., 2012). However, the phyllosphere microecological mechanisms of validamycin are poorly understood, especially its effects on microbial community structure and microbial metabolic function groups, whether on symptomatic or asymptomatic tobacco leaves.

Therefore, the objective of this study was to (i) analyze the structure and diversity of microbial communities in the leaf phyllosphere before and after validamycin application, (ii) investigate the changes of carbon metabolic functions in the phyllosphere before and after validamycin application, and (iii) compare changes in the structure, diversity, and metabolic functions of microbial communities in symptomatic and asymptomatic tobacco leaves after validamycin application.

## Materials and methods

### Experimental design

A field experiment was conducted in commercial tobacco fields of *N. tabacum* cv. Yunyan 105 in August 2020 in Heishitou Town, Weining County, Guizhou Province, China. The region is a humid subtropical monsoon climatic zone. The field was planted with tobacco cultivar Yunyan 105 during 2 years. Two-month old seedlings were transplanted into the field in April each year, and management conditions were typical for tobacco plants. The soil was laterite. Prior to the experiment, tobacco brown spot had sporadically appeared on the tobacco plants in the field, and the incidence rate was about 5–10%. And by the end of the full sampling period, the disease severity was moderate, the incidence rate was more than 20%. On 29 August 2020, nine symptomatic and nine asymptomatic tobacco plants at least 3.5 m apart from each other were randomly selected for first sampling, and tobacco leaves at the same leaf position were collected. In the test, a multifunctional sprayer (model: DSF01A-20-100, Guizhou Qianfengyuan Agricultural Science and Technology Development Co., LTD.) was used to evenly spray the surface of the blade until the droplets disappeared. Then at 5 (September 3), 10 (September 8), and 15 (September 13) days, diseased and healthy tobacco leaves were taken for samples. The classification of tobacco brown spot followed the Chinese National Standard (GB/T 23222–2008) from the Standardization Administration of China.

### Sampling and processing of leaves

Three symptomatic and three asymptomatic tobacco plants were randomly selected from experiment plot, and the middle and lower mature leaves were sampled, respectively. Three strains of tobacco were mixed as one sample and repeated in triplicate. At the time of 0, 5, 10, and 15 days after application, sampling was performed separately. The letters B and J represent diseased and healthy tobacco leaves, respectively (Table 1). All samples were clipped carefully from the leaves using sterile hand shears, and placed into separate 50 mL plastic centrifuge tubes. The tubes were separately packed in sterile zip bags and placed in a portable Styrofoam box with ice packs. Upon arrival in the laboratory, the samples were immediately placed at 4°C for culturing, or at −80°C for DNA extraction for high-throughput sequencing.

**Table 1:** Samples information.

Sampling location		0 day before application	5 days after application	10 days after application	15 days after application
Asymptomatic tissue	Sample No.	J01	J11	J21	J31
		J02	J12	J22	J32
		J03	J13	J23	J33
	Group No.	J0	J1	B2	B3
Symptomatic tissue	Sample No.	B01	B11	B21	B31
		B02	B12	B22	B32
		B03	B13	B23	B33
	Group No.	B0	B1	B2	B3

### Response of microbial communities exposed to validamycin

#### DNA extraction, PCR amplification, and high-throughput sequencing

The DNA of tobacco leaves was extracted by the EasyPure plant genome DNA kit (TransGen Biotech, China) in CTAB method. The solution was visualized using 2% agarose gel electrophoresis, and concentration and purity were assessed by a NanoDrop ND-2000 (Thermo Fisher Scientific, Waltham, MA, United States).

The hypervariable V4 region of bacterial 16S rRNA was amplified with the primers 515F (5′-CCTACGGGRBGCASCAG-3′) and 806R (5′-GGACTACNNGGGTATCTAAT-3′) (Walters et al., 2015). The fungal ITS1 region was amplified using primers ITS5-1F-F (5′-CTTGGTCATTTAGAGGAAGTAA-3′) and ITS1-1F-R (5′-GCTGCGTTCTTCATCGATGC-3′) (Wang et al., 2020). All PCR reactions were carried out with 15 μL of Phusion®High-Fidelity PCR Master Mix (New England Biolabs), 2 μM of forward and reverse primers, and about 10 ng template DNA. Thermal cycling consisted of initial denaturation at 98°C for 1 min, followed by 30 cycles of denaturation at 98°C for 10 s, annealing at 50°C for 30 s, and elongation at 72°C for 30 s, with a final 72°C for 5 min to complete extension. PCR products were purified with the Qiagen Gel Extraction Kit (Qiagen, Germany). Amplification products were used in library construction and sequenced on an Illumina NovaSeq platform targeting 250 bp paired-end reads.

#### Phyllosphere microorganisms sequencing and analysis

Sequences with at least 97% similarity were assigned to the same operational taxonomic unit (OTU) using Uparse software V7.0.1001 (Edgar, 2013). Based on the Mothur algorithm, the taxonomic information of each representative sequence of each OTU was annotated using the UNITE and Silva database. The OTU abundance was normalized to the sample with the fewest sequences, to analyze the alpha and beta diversity of the microbial communities for each sample. The community composition of each sample was counted at the kingdom, phylum, class, order, family, genus, and species levels using the program Unit (V7.24) (Altschul et al., 1990; Kõljalg et al., 2013).

After obtaining the sequencing results and calculation of the OTU matrix, QIIME (V. 1.9.1) was used for full-sample similarity comparison to analyze the alpha diversity and to enumerate taxa. R software (Version 2.15.3) was used to draw dilution curves, rank abundance curves, and species accumulation boxes (White et al., 2009). Beta diversity on both weighted and unweighted unifrac distances was calculated using QIIME software (V.1.9.1). ITS sequences were aligned using Muscle (V.3.8.31) (Edgar, 2004). Phylogenetic analysis was conducted with FastTree 2 (V. 1.9.1) using Maximum Likelihood (ML). Functional composition of fungal and bacterial OTUs was predicted using PICRUSt (Price et al., 2010). Spearman correlation analysis was used to study the relationship between environmental factors and microbial species richness (alpha diversity), and to study relationships between environmental factors (temperature, relative humidity, and rainfall) and species abundance (Algina and Keselman, 1999). The top 50 most abundant species were selected for calculation of correlation coefficients, and correlation coefficients greater than −0.5 and less than 0.5, and *p*-values less than 0.05 were selected for symbiotic network interactions mapping (Qin et al., 2012 Jiao et al., 2017). The FUNGuild database was used to estimate phyllosphere microorganisms trophic mode (Nguyen et al., 2016).

#### Phyllosphere microorganisms carbon metabolism

Fifty Milliliters of 0.8% NaCl (27 mL, w/v) were each mixed with 1 g of tobacco leaves in 100 mL conical flask of different samples (cut into 2 mm x 2 mm pieces), and shaken for 1 h at 28°C. The mixture was then diluted at a ratio of 1:1000 with 0.8% NaCl. Into each of the Biolog Eco - Plates™ (Hayward, California), 150 μL of the dilutions were added and incubated at 28°C in darkness for 7 days in an OmnLog incubator following Deng et al. (2014). The Biolog OmniLog® system (BIOLOG, Hayward, CA, USA) in this study was equipped with a charge-coupled device camera system and an incubator. The color level of each well was measured in OmniLog Units (OU) every 30 min by OmniLog digital camera. The OU values reflected the dynamics of microbial carbon metabolism and represented the utilization of carbon sources. The Biolog ECO plate has 96 microtiter wells, for three replicates per plate. Each of the 32 microtiter wells, contains 1 different organic carbon source and the same amount of tetrazolium violet dye. The organic carbon source in the wells is the only source of energy for the microorganisms, and the tetrazolium violet dye turns purple if the microorganisms can utilize the carbon source after injection into the microtiter wells. The color’s depth reflects the microorganisms’ energy to utilize the carbon source, which indirectly reflects the change in the community composition of microorganisms. Kinetic and Parametric software from Biolog Inc. D5E_OKA_data.exe was used to collect the color intensity data and OL_FM_1.2.exe was used for data analysis” (Cruz et al., 2021), and heat maps were used to analyze the metabolic functions of the samples using HemI software (Baker et al., 2010).

### Statistical analysis

Initial data analysis included taxonomic annotation of OTUs sequences, cluster analysis of all valid sequences of samples using Uparse v7.0.1001 software, annotation analysis, Alpha diversity analysis, Beta diversity analysis, and inter group analysis using Qiime software (Version 1.9.1). IBM SPSS Statistics 23 (IBM Corp., New York, NY, USA) was used to compare the differences of Alpha diversity and Beta diversity indexes of fungal and bacterial communities (*p* < 0.05). The co-occurrence networks of phyllosphere microbial genera and the relationships between environmental factors and microbial genera richness were demonstrated using Spearman’s rank analysis based on significant (*p* < 0.05) and strong positive correlations (*r* > 0.5) or strong negative correlations (*r* < −0.5; Li and Wu, 2018). Statistics were calculated by using Excel 2019, pictures were processed using Adobe Photoshop CS5.

## Results

### Microbial community composition and diversity

**Figure 1:**
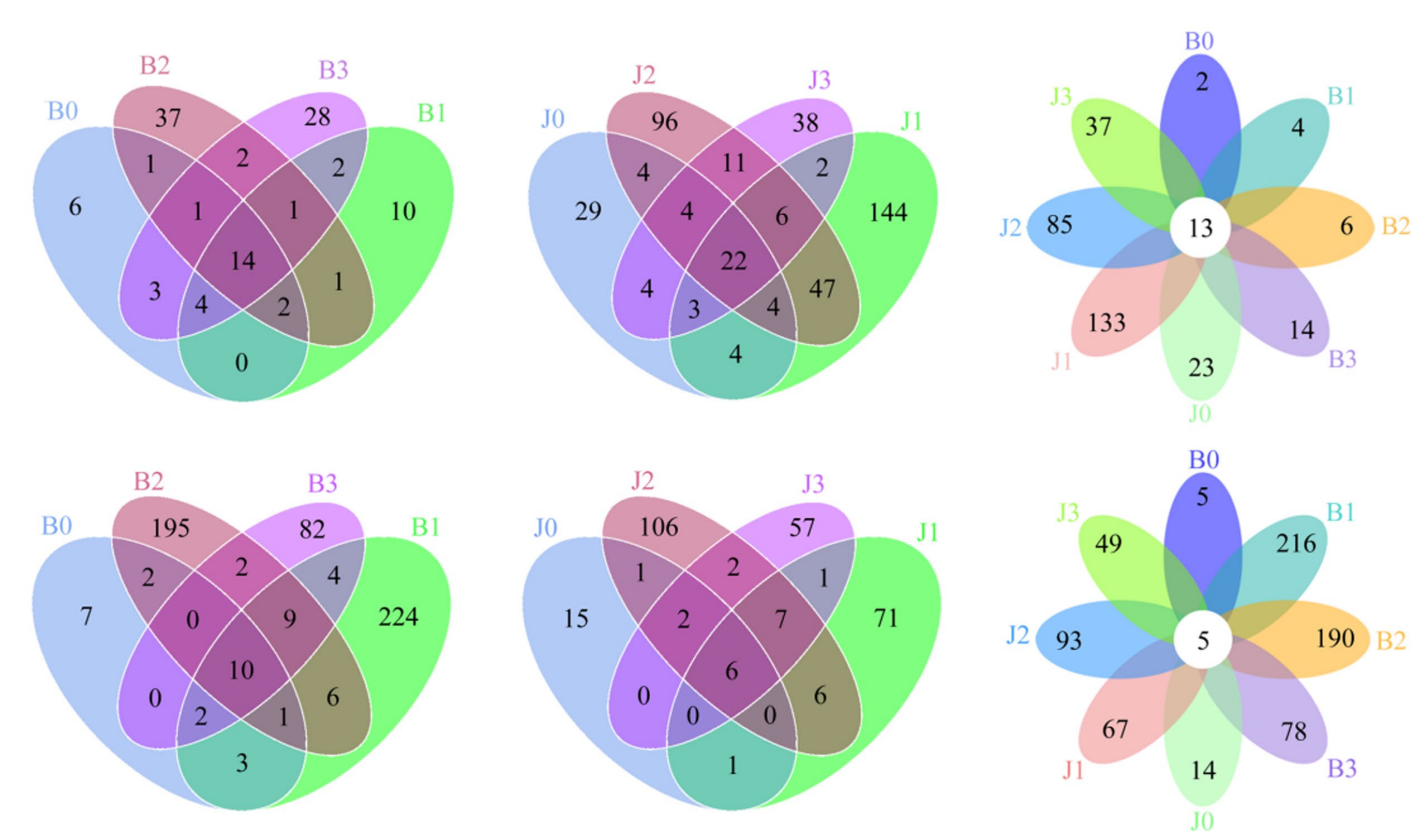
Venn diagram showing the number of microbial OTUs detached in tobacco leaves. **(A–C)** For fungi, **(D–F)** for bacteria. Numbers in the non-overlapping region indicate unique OTUs for the single sample; numbers in the overlapping region indicate shared OTUs for multi-samples; numbers in the core region indicate shared OTUs for each sample.

At the OTUs level, the differences between the communities at symptomatic and asymptomatic leaves were depicted with Venn diagrams. A total of 317 fungal OTUs were discovered, and there were 14, 22, and 13 common fungal OTUs in the symptomatic leaves, asymptomatic leaves, and all leaves, respectively (Figures 1A–C). The OTUs in symptomatic leaves gradually increased after validamycin application, and the OTUs in asymptomatic leaves increased first and then decreased. The number of OTUs was higher in asymptomatic leaves than in symptomatic leaves. A total of 712 bacterial OTUs were discovered in these samples, and there were 10, 6, and 5 core bacterial OTUs for bacterial communities in the symptomatic leaves, asymptomatic leaves, and all leaves at four sampling time points, respectively (Figures 1D–F). The number of OTUs in symptomatic leaves was higher than in asymptomatic leaves. After validamycin application, the independent OTUs numbers in symptomatic and asymptomatic leaves increased and then decreased.

**Figure 2:**
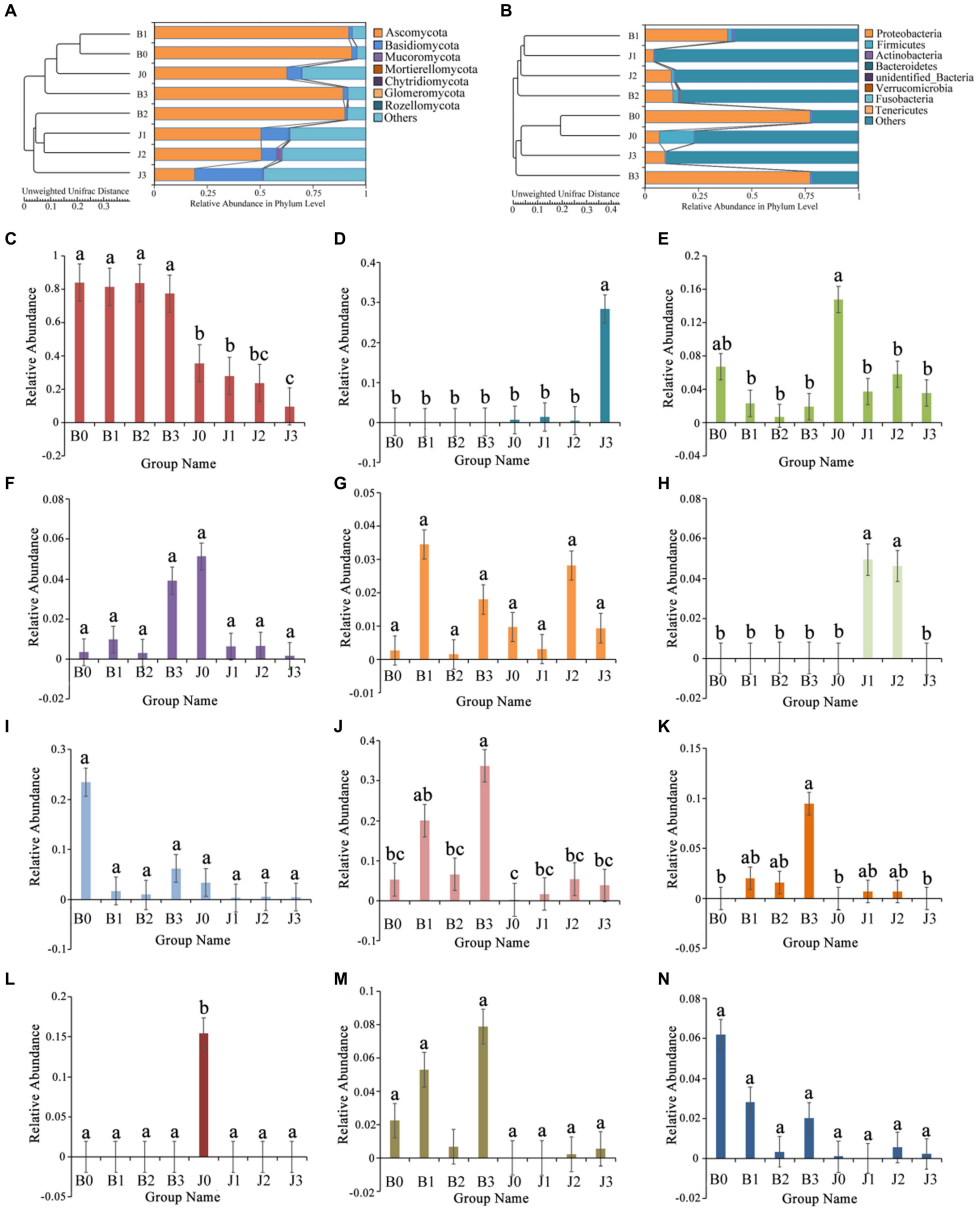
Changes in microbial community (**A** for fungi, **B** for bacteria) compositions at the genus level in tobacco leaves. **(C)** *Alternaria*, **(D)** *Thanatephorus*, **(E)** *Cladosporium*, **(F)** *Boeremia*, **(G)** *Epicoccum*, **(H)** *Archaeorhizomyces*, **(I)** *Kosakonia*, **(J)** *Pseudomonas*, **(K)** *Serratia*, **(L)** *Weissella*, **(M)** *Massilia*, **(N)** *Sphingomonas*. “a, b, and c” represents a statistical difference between groups (*p* < 0.05).

The main fungal phyla were Ascomycota, Basidiomycota, Mucoromycota, Mortierellomycota, and Chytridiomycota (Figure 2A; Table 2). The “Others” fungal group represented all microorganisms that were unidentified or whose relative abundance was less than 0.1% (Figures 2A,B). After validamycin application, the community composition of microorganisms significantly changed, and the relative abundance in symptomatic and asymptomatic leaves decreased for Ascomycota (0.04 and 0.44%) and increased for Basidiomycota (0.01 and 0.25%). At the genus level, the dominant genus in tobacco leaves at the four sampling time points was *Alternaria*, with relative abundances between 77 and 84%, and the relative abundance in asymptomatic leaves was significantly lower than that of symptomatic leaves (Figure 2C). The six most abundant genera were *Alternaria*, *Thanatephorus*, *Cladosporium*, *Boeremia*, *Epicoccum,* and *Archaeorhizomyces* (Figures 2D–H). After validamycin application, the relative abundance of *Thanatephorus* (0.27%) increased and *Archaeorhizomyces* (0.05%) decreased significantly in asymptomatic leaves while *Thanatephorus* was almost absent in symptomatic leaves.

**Table 2:** List of relative abundance in the fungal and bacterial community of the group.

Community Structure			Relative abundance (%)							
			B0	B1	B2	B3	J0	J1	J2	J3
Fungi	Phyla	Ascomycota	0.93 ± 0.05 a	0.92 ± 0.03 a	0.90 ± 0.08 a	0.89 ± 0.03 a	0.63 ± 0.14 b	0.50 ± 0.03 b	0.51 ± 0.15 b	0.19 ± 0.04 c
		Basidiomycota	0.02 ± 0.02 cde	0.02 ± 0.02 de	0.01 ± 0.01 e	0.02 ± 0.01 cde	0.07 ± 0.02 cd	0.13 ± 0.02 b	0.07 ± 0.04 c	0.32 ± 0.06 a
		Mucoromycota	0.00 ± 0.00 a	0.00 ± 0.00 a	0.00 ± 0.01 a	0.00 ± 0.00 a	0.00 ± 0.00 a	0.00 ± 0.00 a	0.02 ± 0.04 a	0.01 ± 0.01 a
	Genera	*Alternaria*	0.84 ± 0.16 a	0.81 ± 0.12 a	0.84 ± 0.07 a	0.77 ± 0.08 a	0.36 ± 0.11 b	0.28 ± 0.02 b	0.24 ± 0.06 bc	0.10 ± 0.03 c
		*Thanatephorus*	0.00 ± 0.00 b	0.00 ± 0.00 b	0.00 ± 0.00 b	0.00 ± 0.00 b	0.01 ± 0.01 b	0.01 ± 0.00 b	0.01 ± 0.01 b	0.28 ± 0.06 a
		*Cladosporium*	0.07 ± 0.10 ab	0.02 ± 0.03 b	0.01 ± 0.00 b	0.02 ± 0.01 b	0.15 ± 0.05 a	0.04 ± 0.05 b	0.06 ± 0.03 b	0.04 ± 0.05 b
		*Boeremia*	0.00 ± 0.00 a	0.01 ± 0.01 a	0.00 ± 0.00 a	0.04 ± 0.05 a	0.05 ± 0.08 a	0.01 ± 0.00 a	0.01 ± 0.00 a	0.00 ± 0.00 a
		*Epicoccum*	0.00 ± 0.00 a	0.03 ± 0.06 a	0.00 ± 0.00 a	0.02 ± 0.02 a	0.01 ± 0.01 a	0.00 ± 0.00 a	0.03 ± 0.02 a	0.01 ± 0.00 a
		*Archaeorhizomyces*	0.00 ± 0.00 b	0.00 ± 0.00 b	0.00 ± 0.00 b	0.00 ± 0.00 b	0.00 ± 0.00 b	0.05 ± 0.02 a	0.05 ± 0.04 a	0.00 ± 0.00 b
Bacteria	Phyla	Proteobacteria	0.38 ± 0.40 b	0.39 ± 0.13 b	0.13 ± 0.08 bc	0.77 ± 0.30 a	0.07 ± 0.10 bc	0.04 ± 0.01 c	0.12 ± 0.06 bc	0.09 ± 0.03 bc
		Firmicutes	0.01 ± 0.01 b	0.02 ± 0.02 b	0.02 ± 0.02 b	0.01 ± 0.01 b	0.16 ± 0.14 a	0.01 ± 0.01 b	0.02 ± 0.01 b	0.01 ± 0.01 b
		Actinobacteria	0.00 ± 0.00 ab	0.01 ± 0.01 a	0.00 ± 0.00 ab	0.00 ± 0.00 b	0.00 ± 0.00 ab	0.00 ± 0.00 b	0.00 ± 0.00 ab	0.00 ± 0.00 b
	Genera	*Kosakonia*	0.23 ± 0.40 a	0.02 ± 0.01 a	0.01 ± 0.00 a	0.06 ± 0.04 a	0.03 ± 0.05 a	0.00 ± 0.00 a	0.01 ± 0.01 a	0.00 ± 0.01 a
		*Pseudomonas*	0.05 ± 0.05 bc	0.20 ± 0.13 ab	0.07 ± 0.09 bc	0.34 ± 0.27 a	0.00 ± 0.00 c	0.02 ± 0.01 bc	0.05 ± 0.01 bc	0.04 ± 0.01 bc
		*Serratia*	0.00 ± 0.00 b	0.02 ± 0.02 ab	0.02 ± 0.00 ab	0.09 ± 0.15 a	0.00 ± 0.00 b	0.01 ± 0.00 ab	0.01 ± 0.01 ab	0.00 ± 0.00 b
		*Weissella*	0.00 ± 0.00 b	0.00 ± 0.00 b	0.00 ± 0.00 b	0.00 ± 0.00 b	0.15 ± 0.13 a	0.00 ± 0.00 b	0.00 ± 0.00 b	0.00 ± 0.00 b
		*Massilia*	0.02 ± 0.04 a	0.05 ± 0.04 a	0.01 ± 0.01 a	0.08 ± 0.12 a	0.00 ± 0.00 a	0.00 ± 0.00 a	0.00 ± 0.00 a	0.01 ± 0.00 a
		*Sphingomonas*	0.06 ± 0.11 a	0.03 ± 0.03 a	0.00 ± 0.00 a	0.02 ± 0.02 a	0.00 ± 0.00 a	0.00 ± 0.00 a	0.01 ± 0.00 a	0.00 ± 0.00 a

There was significant difference between different letters for the data in row direction (*p* < 0.05) according to Duncan’s new multiple range test.

The bacterial OTUs were placed in the Proteobacteria, Firmicutes, Actinobacteria, Verrucomicrobia, and Bacteroidetes, as well as “others” accounting for a large percentage (Figure 2B; Table 2). The relative abundance of Proteobacteria in symptomatic leaves was higher than that in asymptomatic leaves, whereas the relative abundance of Firmicutes on asymptomatic leaves was higher than that on symptomatic leaves. At the genus level, the dominant genera in symptomatic and asymptomatic leaves were *Kosakonia*, *Pseudomonas*, *Serratia*, *Weissella*, *Massilia*, *Sphingomonas*, *Pantoea*, *Erwinia*, *Methylobacterium* and *Akkermansia* (Figures 2I–N). After validamycin application, the relative abundance of *Kosakonia* (0.03%) in asymptomatic tissues was greatly reduced, while those of *Pseudomonas* (0.04%) and *Massilia* (0.01%) in asymptomatic leaves increased. *Weissella* was almost absent on symptomatic leaves, while its relative abundance was higher on asymptomatic leaves, but *Weissella* (0.15%) almost disappeared after the application of validamycin.

### Microbial community alpha diversity

**Figure 3:**
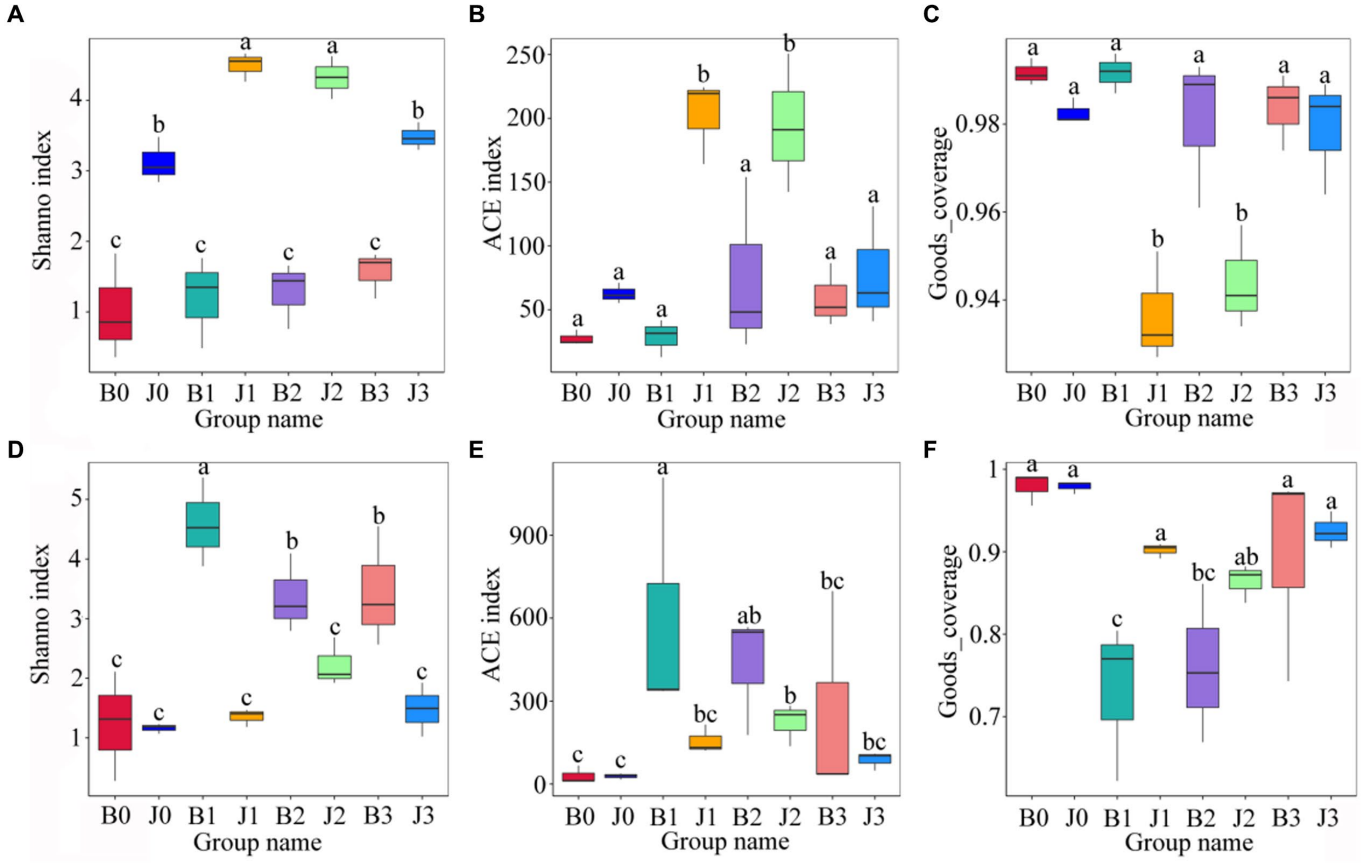
Alpha diversity of the microbial (**A–C** for fungi, **D–F** for bacteria) communities in tobacco leaves. **(A,D)** Show the Shanno index; **(B,E)** show the ACE index; **(C,F)** Show the Goods_coverage. “a, b, and c” represents a statistical difference between groups (*p* < 0.05).

Statistics of Alpha Diversity analysis index (Shannon index, ACE index, goods_coverage) for each sample with 97% threshhold for OTUs were used to assess the differences in species richness and diversity of microbial communities in each sample (Figure 3). Before validamycin application, the Ace index and the Shannon index of fungal communities in asymptomatic leaves were significantly higher than those of symptomatic leaves. At 5 days and 10 days after spraying, these indices of fungal community in asymptomatic leaves increase significantly. There were no significant differences in the Goods_coverage of each sample (Figure 3C).

Significant differences between the Shannon index and the ACE index of bacterial communities in symptomatic and asymptomatic leaves (Figures 3D,E). At 5 days after validamycin application, the Ace index and the Shannon index of bacterial communities increased significantly both in symptomatic leaves and asymptomatic leaves from pre-treatment, and then they decreased significantly.

### Microbial community beta diversity

**Figure 4:**
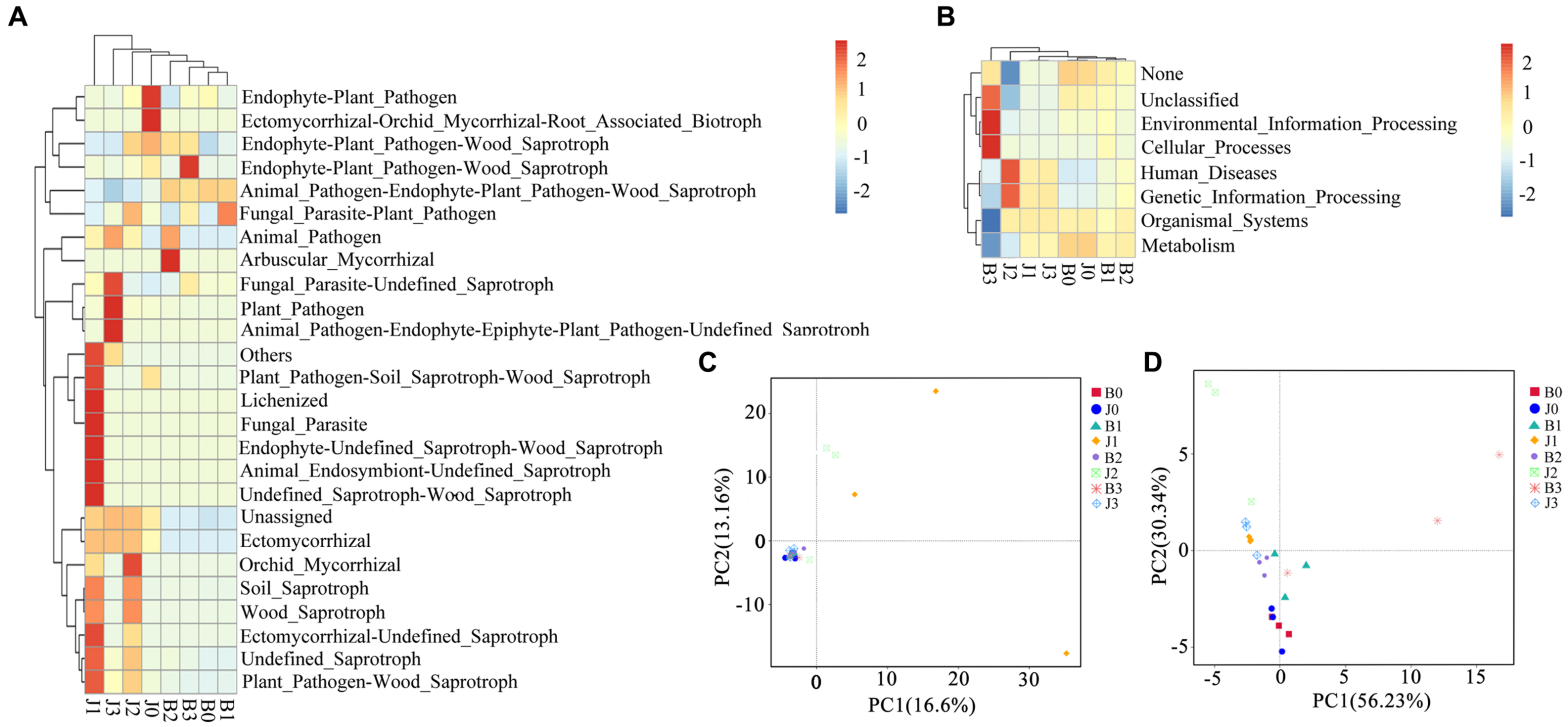
Microbial functional categories and principal coordinate analysis of tobacco leaves. **(A,C)** For fungal communities, **(B,D)** for bacterial communities.

Principal coordinate analysis (PCoA) of the microbial community composition of different samples was based on Unifrac distances. For fungal community composition, nearly all samples were clustered together in the bottom left corner (Figure 4C), and only asymptomatic samples 1 and 2 (out of three replicates) at pretreatment were dispersed on the graph. This indicated that all the symptomatic samples (three each from days 0, 5, 10 and 15) showed little difference in Unifrac distances implying that they share the same OTUs (Figure 4C). The group of J1 and J2 did not cluster with other samples, but J3 had no significant differences from the other samples. The differences between the fungal communities of symptomatic leaves and those of asymptomatic leaves gradually decreased after the application of validamycin.

In the bacterial community composition, most samples were dispersed (Figure 4D). The difference between symptomatic and asymptomatic leaves was more significant, and the difference between after and before application of validamycin was equally significant. Differences between replicate samples of symptomatic and asymptomatic leaves at the same time points after application, and surface validamycin caused a greater effect on the bacterial community composition of symptomatic and asymptomatic leaves.

### Microbial functional prediction

Fungal and bacterial ecological functions were predicted using FUNGuild based on community composition (Figure 4A). For fungal communities, the dominant functions in symptomatic leaves were animal pathogen-endophyte-plant pathogen-wood saprotroph, unassigned, plant pathogen, endophyte-plant pathogen, undefined saprotroph. In asymptomatic leaves in the fungal community, the dominant functions were animal pathogen-endophyte-plant pathogen-wood saprotroph, the dominant functions in symptomatic leaves were unassigned, followed as endophyte-plant pathogen, undefined saprotroph. Asymptomatic leaves were richer in fungal functional groups than that of symptomatic leaves. Asymptomatic leaves had more ecologically functional groups than symptomatic leaves for fungal communities.

Bacterial families were estimated using PICRUSt (Figure 4B). The diversity of functional groups of bacteria in asymptomatic and symptomatic leaves was significantly lower than that of fungi. In this study, metabolism, genetic information processing, unassigned, environmental information processing, and cellular processes were the dominant bacterial community functions common to all samples, only differing in their relative abundance from sample to sample. The functional groups of B3 and J2 were relatively more concentrated in distribution.

### The relationship between microorganisms and environmental conditions

**Figure 5:**
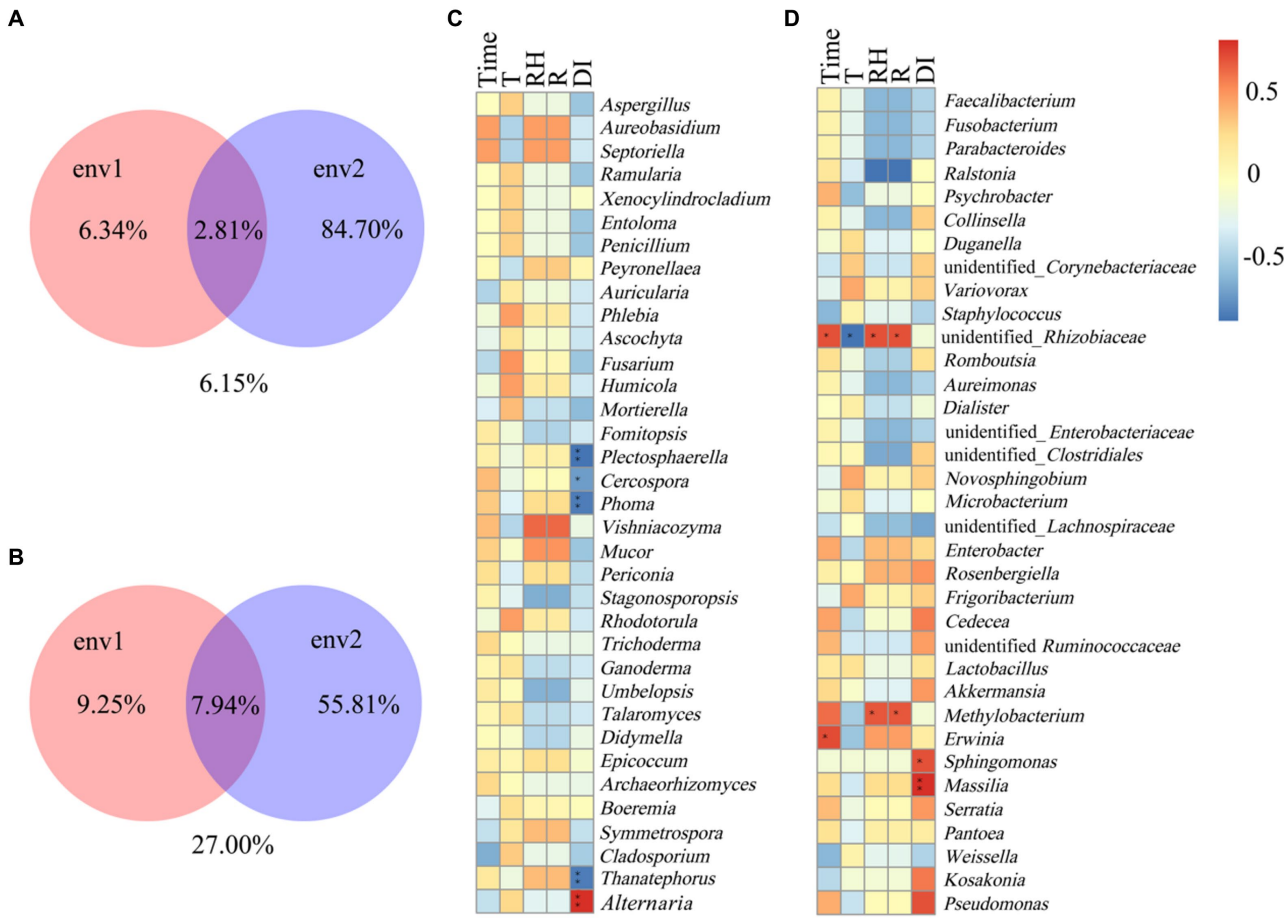
Spearman correlation analysis between microbial communities and environmental factors in tobacco leaves. **(A,C)** For fungal communities, **(B,D)** for bacterial communities. Temperature (T), relative humidity (RH), rainfall (R), disease index (DI). “*” Represents a statistical difference between groups (*p* < 0.05), “**“represents a significant statistical difference between groups (*p* < 0.01) (env1: Time, T, RH; env2: R, DI).

The Spearman correlation coefficient was used as a measure to study the relationship between environmental factors (time, temperature, relative humidity, rainfall) and microorganisms. Variance Partitioning analysis was used to account for the proportion of different environmental factors explaining changes in microbial community occurrence (Figure 5). Env1 included time, temperature, env2 included rainfall and disease index. In the fungal community, env2 occupied 84.70% of the community structure and env1 occupied 6.34% of the fungal community structure (Figure 5A). For fungal communities, the disease index was significantly and positively correlated with *Alternaria*, while negatively correlated with *Thanatephorus*, *Phoma*, *Cercospora*, and *Plectosphaerella* (Figure 5C). In the bacteria community, env2 occupied 55.81% of the community structure and env1 occupied 9.25% of the community structure (Figure 5B). Time was significantly and positively correlated with unidentified*_Rhizobiaceae* and *Erwinia*, the temperature was negative correlated with unidentified*_Rhizobiaceae*, relative humidity, and rainfall were positively correlated with unidentified*_Rhizobiaceae* and *Methylobacterium*, and disease index was significantly and positively correlated with *Sphingomonas* and *Massilia* (Figure 5D).

### Co-occurrence network analysis between microorganisms

**Figure 6:**
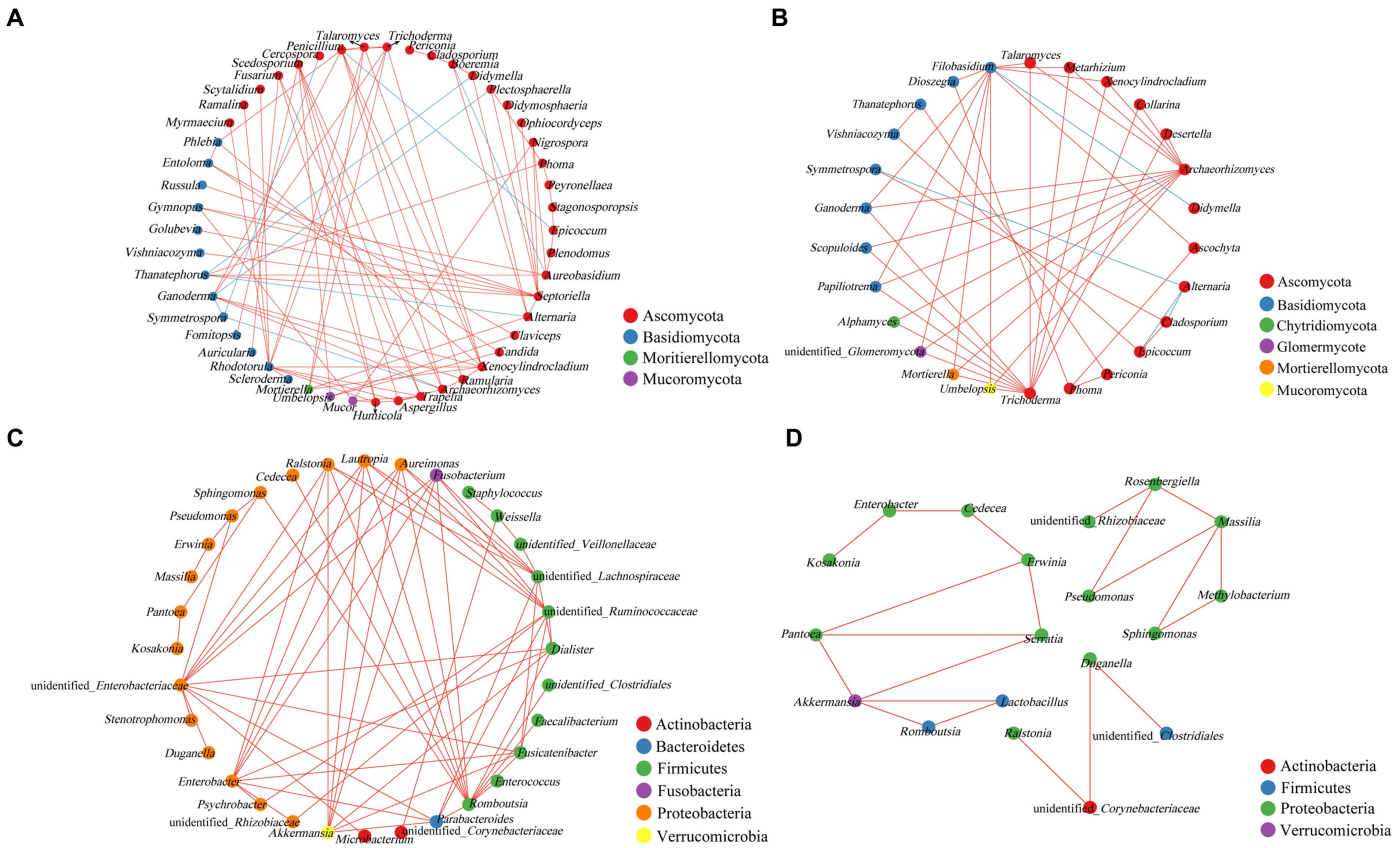
Co-occurrence networks of microbial communities at genus level. **(A,B)** For fungal communities, **(C,D)** for bacterial communities (**A,C** for asymptomatic leaves, **B,D** for symptomatic leaves). Node color represents the phylum of the genus and the size corresponds to the centrality score. Red lines indicate positive relationships, and blue lines indicate negative relationships.

Network analysis of the co-occurrence of the most abundant 50 fungal species revealed that 49 fungal species were showing positive co-occurrence with each other (*p* < 0.05, *r* < −0.5). In fungal genera, 8 strong negatives and 4 strong negative correlations were identified from asymptomatic and symptomatic leaves, respectively (Figures 6A,B). As the pathogen of tobacco brown spot, the positively correlation was showed between *Alternaria* with *Mortierella*, *Boeremia,* and *Didymella*, and negatively correlated with *Thanatephorus* and *Septoriella*. In asymptomatic leaves, *Thanatephorus* showed positive co-occurrence with *Didymella*, *Epicoccum* with *Penicillium*, and *Ganoderma* with *Plectosphaerella* and *Symmetrospora*. *Alternaria* was positively correlated with *Symmetrospora*, *Epicoccum*, and *Cladosporium* in asymptomatic leaves, and *Didymella* was positively correlated with *Filobasidium*. There were only positive correlations between bacterial genera (Figures 6C,D).

### Microbial community carbon metabolic profiles

**Figure 7:**
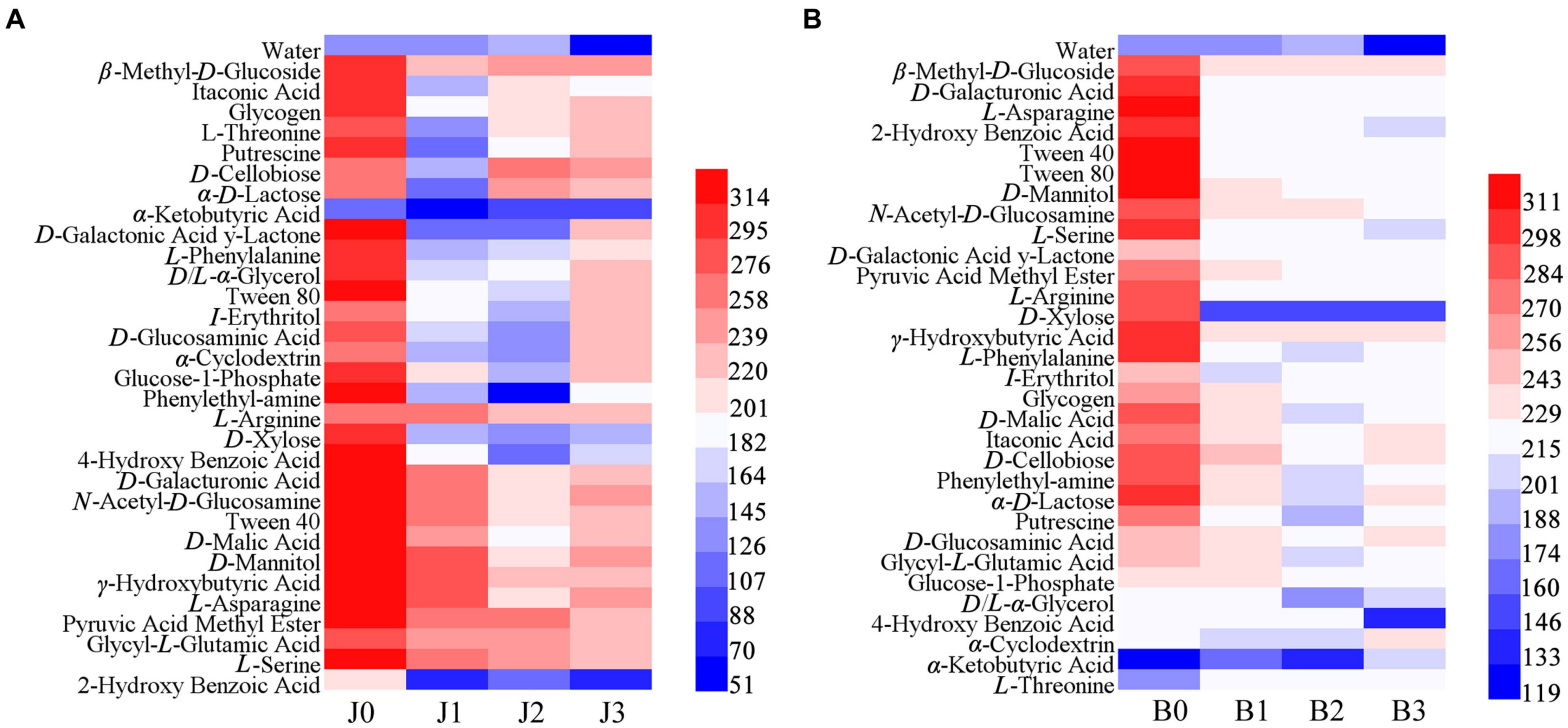
Cluster heat map carbon sources in Biolog ECO-Plate of microbial communities from different samples (**A** for asymptomatic leaves, **B** for symptomatic leaves).

The Biolog ECO microplate was used to explore the metabolism of 31 basal carbon sources in symptomatic and asymptomatic leaves after validamycin application (Figure 7). The results showed that microbial communities were more capable of utilizing carbohydrates before validamycin, the metabolic capacity was reduced after the agent treatment. In asymptomatic tissues, *L*-arginine, *γ*-hydroxybutyric acid, and pyruvic acid methyl ester had strong metabolic capacity, *α-*keto butyric acid, and 2-hydroxy benzoic acid had lower metabolism. The metabolic capacity of asymptomatic tissues for 2-hydroxy benzoic was significantly reduced after validamycin treatment, with most metabolic capacities rising before decreasing. The metabolism of carbon sources from symptomatic was significantly reduced after the drug treatment. Reduced metabolism of *D*-xylose, 4-hydroxy benzoic acid, and *D*/*L*-*α*-glycerol and increased metabolism of *α*-cyclodextrin, *α*-ketobutyric acid and *L*-threonine in symptomatic leaves after validamycin treatment.

## Discussion

Field application of validamycin (0.11 Kg a.i./ ha^−1^) presented a higher effect on *Alternaria* on asymptomatic leaves than that of symptomatic leaves. In our study, validamycin had significant effects on the phyllosphere microorganisms of tobacco leaves. After the application of validamycin, the relative abundance of *Alternaria* decreased on both symptomatic leaves and asymptomatic leaves. The relative abundance of *Alternaria* was reduced more on asymptomatic leaves. When 50% Kresoxim-methyl was used, the relative abundance of *Alternaria* was reduced in both healthy and diseased leaves, but the effect of the agent was greater on healthy leaves than on diseased leaves (Xiang et al., 2022). In studies on the phyllosphere microorganisms at different stages after the onset of tobacco brown spot, the relative abundance of *Alternaria* increased with the degree of disease (Dai et al., 2022). They found that validamycin can control the relative abundance of the pathogen at the onset of the tobacco brown spot. Validamycin had limited effect in the treatment of existing tobacco brown spot, but the relative abundance of *Alternaria* in asymptomatic leaves can be effectively suppressed. We recommend that validamycin be used preventively and not curatively. However, quantitative measurements of the microorganisms and more field trials on validamycin are still needed to further determine the efficacy of validamycin.

The species composition of phyllosphere microorganisms in tobacco changed after validamycin application. The species richness and community diversity of phyllosphere microorganisms increased at the beginning but decreased with the increase in application time. This implies that validamycin can inhibit the increase in the relative abundance of potential pathogens on asymptomatic leaves quickly, but low persistence and multiple applications can be considered if using validamycin for disease prevention. In a study of phyllosphere microorganisms in tobacco brown spot, the species richness and community diversity of fungi were higher in asymptomatic leaves than in symptomatic leaves, while the species richness and community diversity of bacteria were higher in asymptomatic leaves than in symptomatic leaves (Xiang et al., 2022). The dominant fungal genera were *Alternaria*, *Thanatephorus*, *Cladosporium*, *Boeremia*, and *Epicoccum*, and the dominant bacterial genera were *Kosakonia*, *Pseudomonas*, and *Serratia*. In previous studies, tobacco’s dominant fungi and bacteria were *Alternaria*, *Didymella*, *Cladosporium*, *Pseudomonas* and *Sphingomonas*, respectively. There were slight differences in the results of the two studies. However, in addition to tobacco target spot pathogens, the dominant genera were equally high in *Alternaria*, *Pseudomonas* and *Sphingomonas* during the onset of tobacco target spot (Sun M. L. et al., 2023). This indicates that the four fungi can exist together and even affect each other’s disease development. However, *Pseudomonas* are a group of bacteria that are widely found in the environment, plants, and air. *Pseudomonas syringan* pv. tabaci and *P. syringae* pv. angula are the causal agents of tobacco wildfire disease and tobacco bacterial angular spot disease, respectively, that can cause bacterial diseases of tobacco (Turner et al., 2020). *Sphingomonas,* a non-pathogenic bacterial genus, shows a strong antagonistic effect against *Diaporthecitri* on citrus (Li et al., 2022), but its relative abundance on asymptomatic leaves was significantly reduced by validamycin. *Thanatephorus* and *Archaeorhizomyces* were relatively abundant on symptomatic leaves but almost absent on asymptomatic leaves. *Cladosporium*, *Kosakonia*, *Pseudomonas*, and *Sphingomonas* were present on both symptomatic and asymptomatic leaves. These bacteria were also found in phyllosphere microbial investigations of tobacco leaf spot caused by *Didymella* and tobacco leaf mildew and rot (Huang Z. et al., 2021), implying that these bacteria may be resident on tobacco leaves. Validamycin application reduced the relative abundance of *Cladosporium*, *Kosakonia* and *Sphingomonas* in addition to the pathogen, *Alternaria*. *Cladosporium* as a potential pathogen, can also cause diseases in some crops such as cucumber, tomato, and canola (Batta, 2004; Rivas and Thomas, 2005 Idnurm et al., 2021). Guo et al. (2023) explored the effect of azoxystrobin on phyllosphere microorganisms of tobacco brown spot, and showed a similar decrease in the relative abundance of *Cercospora* in addition to *Thanatephorus*, but an increase in the relative abundance of *Pseudomonas* and *Sphingomonas*. When the fungicide STROBY (containing azoxystrobin) was used to control tobacco brown spot, the agent caused a decrease in community diversity in symptomatic leaves and an increase in bacterial community diversity, with STROBY having a significantly greater effect on the fungal community than on the bacterial community (Xiang et al., 2022). When agents are applied for disease control, they affect the entire phyllosphere microecology, and act on multiple pathogenic or beneficial bacteria at the same time, resulting in changes in the structure and diversity of phyllosphere microorganisms to achieve control of disease exacerbation (Wang et al., 2016).

The metabolic capacity of asymptomatic leaves remained higher than that of symptomatic leaves after validamycin application, and both had a lower metabolic capacity for 2-hydroxy benzoic. The results are the same with those of the metabolic capacity for 2-hydroxy benzoic in studies on the metabolism of phyllosphere microorganisms of tobacco brown spot at different spatiotemporal environments (Dai et al., 2022). And 2-hydroxy benzoic also named salicylic acid, it was closely related to plant immunity and disease resistance (An and Mou, 2011). However, further experiments are needed to determine whether this metabolic decline was due to changes in phyllosphere microorganisms. After validamycin, the metabolic capacity of symptomatic and asymptomatic leaves was significantly reduced. But 15 days after the application, the metabolic capacity of asymptomatic tobacco leaves started to recover. It is possible that the number of phyllosphere microorganisms decreased and their metabolic capacity for carbon sources was reduced since the agents significantly controlled the relative abundance of the pathogenic *Alternaria* already present in asymptomatic tobacco leaves. This result was similar to the results of phyllosphere microbial metabolism capacity after Bordeaux mixture for tobacco target spot control (Li et al., 2022). But afterward, the community diversity started to recover, the diversity increased and the metabolic capacity for carbon sources started to recover. In contrast, the asymptomatic tissues were unable to recover their metabolic capacity, because the integrity of the tobacco leaf was damaged from the onset of the disease and the relative abundance of *Alternaria* was too high. The relative abundance of beneficial bacteria could not be restored to normal values, Phyllosphere microbes unable to restore homeostasis. This is important for plant leaves to stay healthy (Liu et al., 2020).

The relationship between different microorganisms on the tobacco leaves was showed in the co-occurrence network (Deng et al., 2012; Vacher et al., 2016). In this study, there were significantly more interactions between fungi than bacteria, and the interrelationships between asymptomatic leaves were stronger than those of symptomatic leaves. The results were in common with the variation in the relative abundance of species, with negative correlations between *Alternaria* and *Thanatephorus*. In Spearman correlation analysis, the disease index was significantly and positively correlated with *Alternaria*, while negatively correlated with *Thanatephorus*, *Phoma*, *Cercospora*, and *Plectosphaerella*. The two results suggest a possible competitive relationship between *Alternaria* and *Thanatephorus*, which was found to be similarly negatively correlated with *Alternaria* and *Thanatephorus* after validamycin application in a study of tobacco target spot disease (Guo et al., 2023). In tobacco leaves, *Alternaria* and *Thanatephorus* may have a competitive relationship, the two are different tobacco disease pathogens, when the disease occurs at the same time, the pathogen proliferation generously, the environment, nutrients, the same increase in the demand for habitation, the leaf resources are limited, one of them first increase, will inhibit the growth of the other pathogen, and the relative abundance of the non-pathogenic bacteria is low, the energy required is less, will not be affected from it too much.

After validamycin application, the relative abundance of *Alternaria* on asymptomatic leaves decreased and the relative abundance of *Thanatephorus* increased significantly. The proliferation of pathogenic bacteria led to a dramatic increase in the demand for space and nutrients in the leaves, and the proliferation of one of them inhibited the growth of the other. The interactions between the bacteria showed positive correlations, which may be related to the population abundance of each pathogen, or it may be that the stronger effect of validamycin on phyllosphere bacteria directly caused the relative abundance of a large number of bacteria to decrease, but the competition, fitness, and environmental adaptability of these pathogens when coexisting deserve further study. Meanwhile, this experiment provides a limited reference for the control of tobacco target spot disease by validamycin. We still need further field investigations on the specific efficacy of validamycin, and quantitative analysis of phyllosphere microorganisms to gain a deeper understanding of the control effects.

## Conclusion

The results of this study showed that the dominant phyllosphere microorganisms of tobacco brown spot were *Alternaria*, *Thanatephorus, Kosakonia*, and *Pseudomonas*, while phytopathogenic fungi such as *Boeremia*, and *Epicoccum* were also present in the leaves. The community diversity indices of fungi and bacteria of symptomatic and asymptomatic leaves increased after the application of validamycin, and the microbial community diversity of asymptomatic leaves was higher than that of symptomatic leaves. Microbial community richness increased and then decreased in both symptomatic and asymptomatic leaves. The effect of validamycin on the fungal community on asymptomatic leaves was greater than that on symptomatic leaves, and the structure of the bacterial community on symptomatic leaves was greater than that on asymptomatic leaves. The effect of validamycin on phyllosphere microorganisms gradually decreased with increasing application time.

## Data availability statement

The datasets presented in this study can be found in online repositories. The names of the repository/repositories and accession number(s) can be found a: https://www.ncbi.nlm.nih.gov/, PRJNA982433.

## Author contributions

MG: Data curation, Formal analysis, Investigation, Methodology, Project administration, Software, Validation, Writing – original draft. JH: Conceptualization, Supervision, Writing – review & editing. CJ: Conceptualization, Supervision, Writing–review & editing. YZ: Data curation, Project administration, Writing – review & editing. HW: Conceptualization, Funding acquisition, Resources, Supervision, Writing – review & editing. XZ: Data curation, Project administration, Writing – review & editing. TH: Methodology, Supervision, Writing – review & editing. CS: Conceptualization, Writing – review & editing. QW: Data curation, Project administration, Writing – review & editing. FW: Resources, Writing – review & editing.
